# Deciphering the Bacterial Microbiome in Huanglongbing-Affected Citrus Treated with Thermotherapy and Sulfonamide Antibiotics

**DOI:** 10.1371/journal.pone.0155472

**Published:** 2016-05-12

**Authors:** Chuanyu Yang, Charles A. Powell, Yongping Duan, Robert Shatters, Jingping Fang, Muqing Zhang

**Affiliations:** 1 State Key Lab for Conservation and Utilization of Subtropical Agro-biological Resources, Guangxi University, Nanning, 530005, China; 2 Indian River Research and Education Center, IFAS, University of Florida, Fort Pierce, FL, 34945, United States of America; 3 Horticultural Research Lab, USDA-ARS, Fort Pierce, FL, 34945, United States of America; 4 College of Crop Science, Fujian Agriculture and Forestry University, Fuzhou, 350002, China; Fujian Agriculture and Forestry University, CHINA

## Abstract

Huanglongbing (HLB) is a serious citrus disease that threatens the citrus industry. In previous studies, sulfonamide antibiotics and heat treatment suppressed ‘*Candidatus* Liberibacter asiaticus’ (Las), but did not completely eliminate the Las. Furthermore, there are few reports studying the bacterial microbiome of HLB-affected citrus treated by heat and sulfonamide antibiotics. In this study, combinations of heat (45°C or 40°C) and sulfonamide treatment (sulfathiazole sodium–STZ, or sulfadimethoxine sodium—SDX) were applied to HLB-affected citrus. The bacterial microbiome of HLB-affected citrus following thermotherapy and/or chemotherapy was characterized by PhyloChip^TM^G3-based metagenomics. Our results showed that the combination of thermotherapy at 45°C and chemotherapy with STZ and SDX was more effective against HLB than thermotherapy alone, chemotherapy alone, or a combination of thermotherapy at 40°C and chemotherapy. The PhyloChip^TM^G3-based results indicated that 311 empirical Operational Taxonomic Units (eOTUs) were detected in 26 phyla. *Cyanobacteria* (18.01%) were dominant after thermo-chemotherapy. Thermotherapy at 45°C decreased eOTUs (64.43%) in leaf samples, compared with thermotherapy at 40°C (73.96%) or without thermotherapy (90.68%) and it also reduced bacterial family biodiversity. The eOTU in phylum *Proteobacteria* was reduced significantly and eOTU_28, representing “*Candidatus* Liberibacter,” was not detected following thermotherapy at 45°C. Following antibiotic treatment with SDX and STZ, there was enhanced abundance of specific eOTUs belonging to the families *Streptomycetaceae*, *Desulfobacteraceae*, *Chitinophagaceae*, and *Xanthomonadaceae*, which may be implicated in increased resistance to plant pathogens. Our study further develops an integrated strategy for combating HLB, and also provides new insight into the bacterial microbiome of HLB-affected citrus treated by heat and sulfonamide antibiotics.

## Introduction

Citrus species are important fruit trees worldwide. Currently, citrus huanglongbing (HLB) is a destructive disease for the citrus industry worldwide, and has caused severe economic losses for the citrus industries in American, Asian and African nations [1, 2]. For instance, Florida's $10 billion citrus industry is an important sector of the state economy, providing thousands of jobs and capital to agricultural regions [3]. Florida produces 59% of the total U.S. citrus supply, but diseases like citrus HLB are threatening this production [3]. HLB has been responsible for $3.63 billion in lost revenues and 6,611 lost jobs in the orange juice production industry alone since 2006 [4].

HLB is caused by 3 species of fastidious, phloem-restricted α-proteobacteria: ‘*Candidatus* Liberibacter asiaticus’ (Las), ‘*Candidatus* Liberibacter americanus,’ and ‘*Candidatus* Liberibacter africanus’ [1, 5, 6] and is transmitted by insect vectors *Diaphorina citri* or *Trioza erytreae* [7]. Of these 3 species, Las is the one currently affecting citrus in the USA. Although HLB-resistant citrus varieties are being developed to combat the disease, it will likely be many years before they are available. Chemotherapy has shown considerable promise for control of HLB in the short term. In our previous study, several antibiotics effective against Las were evaluated by a graft-based method and sulfonamide antibiotics were highly effective at reducing Las titers [8]. Sulfonamide antibiotics have been reported to improve the root morphology and functionality in barley [9]. The root system of HLB-affected citrus trees is often poorly developed and new root growth may be suppressed [10]. Thus, we hypothesize that sulfonamide antibiotics have potential for controlling HLB by root drench application.

Thermotherapy is also an effective strategy against HLB and can enhance the vigor of citrus and promote new root growth [11]. Las, which is a heat-tolerant bacterium, can thrive at temperatures as high as 35°C [12]. Many studies have demonstrated that HLB-affected citrus can recover and Las titers can be suppressed or eliminated by thermotherapy above 40°C [11, 13, 14]. However, HLB is a systemic disease and effective elimination of the bacterium from the entire citrus tree, including roots, is essential for a long-term cure. To overcome this barrier, thermotherapy combined with chemotherapy may be a very efficient strategy for control of HLB.

The microbial communities of leaves are diverse and bacteria are the most abundant inhabitants [15]. It has been suggested that cell density-dependent signaling may play a role in epiphytic bacterial fitness [15]. Bacterial cells in close proximity may be able to modify their microenvironment, which could make the composition of the microbial community a key factor in the ability of Las to cause HLB progression [16, 17]. The microbial diversity associated with citrus HLB in planta has been revealed by several recent works [17–20]. In our previous research, changes in the bacterial microbiome of HLB-affected citrus in response to antibiotics such as ampicillin, gentamicin, a combination of penicillin and streptomycin, and a combination of kasugamycin and oxytetracycline were deciphered by PhyloChip^TM^G3 array [16, 21]. Several reports have concluded that sulfonamide antibiotics can affect both the function and structural diversity of the soil community [22–24]. In addition, the composition of the microbial community in soil was affected by different temperature treatments [25]. However, the microbiome of HLB-affected citrus in response to heat and sulfonamide treatment has been poorly studied. We hypothesize that variation of the microbiome in response to sulfonamide antibiotics and thermotherapy may be deleterious to Las. In this paper, we aim to decipher changes in the bacterial microbiome in HLB-infected citrus after thermotherapy at various temperatures and chemotherapy with sulfonamide antibiotics.

## Materials and Methods

### Plant materials

Two-year-old healthy grapefruit (*Citrus paradisi*) seedlings were graft-inoculated with HLB-infected lemon (*Citrus*. *limon*) scions. Infection was confirmed by quantitative real-time polymerase chain reaction (qPCR) and plants were subsequently maintained in a greenhouse. After 10 months, typical HLB symptoms such as vein corking and blotchy mottles appeared on the leaves of the inoculated seedlings. Seedlings with typical HLB symptoms were then tested by qPCR as described below for the presence of Las using a Las-specific primer/probe set [26].

### Thermo-chemotherapy

HLB-infected grapefruit seedlings were treated by thermotherapy at 40°C or 45°C in growth chambers for 1 week and then returned to room temperature 25°C (RT) in a greenhouse. Non treated controls remained in the greenhouse. Growth chambers were set as follows: constant 85% relative humidity; midnight– 8 am, dark at 25°C, 8 am -10 am, light at 35°C, 10 am to 10 pm, light at 40°C or 45°C (thermotherapy for 12 h), 10 pm to midnight, dark at 25°C. Citrus seedlings were also treated with 2 L 500 mg/L sulfadimethoxine sodium (SDX) (obtained from Sigma-Aldrich, St. Louis, MO), or 2 L 500 mg/L sulfathiazole sodium (STZ) (obtained from Sigma-Aldrich, St. Louis, MO), using root drench application twice during one month. A 2 L tap water drench served as the non-treated control (CK). Three HLB-affected citrus seedlings were in each treatment group. Five leaf samples were collected and mixed at 0, 60, 180 and 300 days after the initial chemical treatment in each HLB-affected citrus seedling, then used for DNA extraction and qPCR analysis.

The resulting Ct values were converted to estimated bacterial titers using a regression equation Y = 13.82–0.2866X, where Y is the estimated log concentration of template and X is the Ct value from qPCR, as described by Li et al. [26]. The area under the disease progress curve standardized (AUDPCs) per treatment was calculated using Ct values from day 0 to day 300. Las titer decreases as Ct value increases, so the AUDPC increases as Las titer is reduced. HLB disease severity was assigned a grade from 0 to 4, where 0 = Ct value ≥ 36.0 (Las undetectable), 1 = 32.0 ≤ Ct value < 36 (Low Las titer), 2 = 28 ≤ Ct value < 32 (Moderate Las titer), 3 = 24 ≤ Ct value < 28 (High Las titer), and 4 = Ct value < 24 (Very high Las titer). The disease index (DI) was calculated as $\text{DI}=\frac{Sum\;of\;all\;numerical\;grades}{Total\;number\;of\;plants\;counted\times maximum\;grade}\times 100$.

### Genomic DNA extraction and qPCR analysis

Leaves for DNA extraction and analysis were rinsed three times with sterile water. Midribs were separated from the leaf samples and cut into 1.0 to 2.0 mm pieces. DNA was extracted from 0.1 g (fresh weight) of leaf midrib tissue using a DNeasy Plant Mini Kit (Qiagen, Valencia, CA) according to the manufacturer’s protocol. qPCR was performed with primers and probe (HLBas, HLBr and HLBp) specific for Las [26] using the ABI 7500 Fast Real-Time PCR System (Applied Biosystems, Foster City, CA) in a 20 μl reaction volume consisting of the following reagents: 300 nM (each) target primer (HLBas and HLBr), 150 nM target probe (HLBp), and 1× TaqMan qPCR Mix (Applied Biosystems). The amplification protocol was 95°C for 20 s followed by 40 cycles at 95°C for 3 s and 60°C for 30 s. All reactions were performed in triplicate and each run contained one negative (DNA from healthy plant) and one positive (DNA from Las-infected plant) control. Data were analyzed using the ABI 7500 Fast Real-Time PCR System with SDS software.

### PCR amplification of 16S rRNA genes and PhyloChip^TM^ G3 analysis

DNA for the PhyloChip^TM^G3 analysis was extracted at 300 days after the initial chemical treatment as described above. DNA from five leaves in each treatment was pooled in equal amounts of three replicates and quantified by the PicoGreen method (Life Technologies, Grand Island, NY). PCR amplifications of 16S rRNA genes were conducted as described previously [16, 21]. The PhyloChip^TM^G3 analysis was carried out by Second Genome Inc. (San Bruno, CA) using the recommended protocol for Affymetrix Prokaryotic Arrays as described in previous studies with some modifications [16, 21, 27, 28]. Array fluorescence intensity (FI) of each pixel in an image was collected as integer values ranging from 0 to 65,536 providing 216 distinct FI values. The summary of FI for each single probe feature on the array was calculated by ranking the FI of the central 9 of 64 image pixels and using the value of at 75%. Background was defined as the mean feature FI in the least bright 1% of features in each of 25 equally-divided sectors of the image. The background was subtracted separately in each sector. Next, all probes on the array were scaled by multiplication with a single factor so that average FI of the probes perfectly matching the PhyloChip^TM^G3 Control Mix of non-16S spikes (part # 49-0001A) were equal. FI values from redundant probes were averaged to generate the simple probe-level table representing the responses of 994,980 25mers across all samples. Pairs of probes are two probes with similar but non-identical sequences which align along > = 23 bases with > = 1 mismatch or gap as determined by blast [29]. Although all probes can produce minor fluorescence from non-specific hybridization, if a sequence-specific hybridization has occurred the probe complementing the target will be brighter than its mis-matching mate as has been observed in 70% of controlled experiments [30]. As a general caution, perfect matching probes (PM) were considered positive if they fulfilled the following criteria in comparison to their corresponding mis-matching probes (MM). A) PM/MM > = 1.5, B) PM-MM > = 50*N and r > = 0.95, where N is the array specific noise, and r indicates the response score [27].

PM fluorescence intensities from 994,980 probes observed as “positive” were exported, then ranked within each sample and used as input to empirical probe-set discovery by cluster analysis. Probes were clustered into probe-sets or an empirical Operation Taxonomic Unit (eOTU) based on both correlations in fluorescence and intensities across all biological samples and taxonomic relatedness. All probe sets contained ≥ 5 probes with average pair-wise correlation coefficients ≥ 0.60 (*p* < 0.05, 10 degrees of freedom). The eOTU tracked by a probe set was taxonomically annotated from the combination of the 8-mers contained in all probes of the set [31]. Where standard taxonomic names have not been established, hierarchical taxon identifier was used (for example “94OTU36152”). The mean FI for each eOTU and each sample was calculated and then rank-normalized within each sample. These values are referred to as the hybridization score (HybScore) used in abundance based analysis. After the taxa are identified for inclusion in the analysis, the values used for each taxa-sample intersection are populated in two distinct ways: i) Abundance metrics were used directly (AT) and hybridization score (Hybscore) used in analysis; ii) Binary metrics were created where 1’s represent presence and 0’s indicate absence (BT).

The eOTUs were filtered to taxa significantly increased in abundance in one category compared to the alternate categories (Filter-5). For Filter-5, the Welch test was employed to calculate p-values. Additionally, q-values were calculated using the Benjamini-Hochberg procedure to control for false discovery rates in multiple testing. For weighted Unifrac or WUnifrac, the eOTUs abundance is considered. For BT, Unifrac was used; whereas, for AT, WUnifrac was used. Principal Coordinate Analysis (PCoA) is a method of two-dimensional ordination plotting that is used to visualize complex relationships between samples. PCoA uses the dissimilarity values to position the points relative to each other.

### Bacterial biodiversity index

The Shannon and Simpson biodiversity indices combine components of species number and relative abundance [32]. In this paper, they were used to analyze the differences in bacterial diversity in response to thermo-chemotherapy calculated from present eOTUs as: Shannon’s index = -$\sum_{1}^{\text{n}}F\text{i}$*LnFi* and Simpson’s diversity index = 1 -$\sum_{1}^{\text{n}}{F\text{i}}^{2}$. Here, n represents the richness or total number of families, *Fi* is the proportion of the present OTUs accounted for by the *i*^th^ phylum from the total eOTUs detected, and *Ln* is the natural logarithm.

## Results

### Effect of thermotherapy and chemotherapy on control of citrus HLB

**Fig 1:**
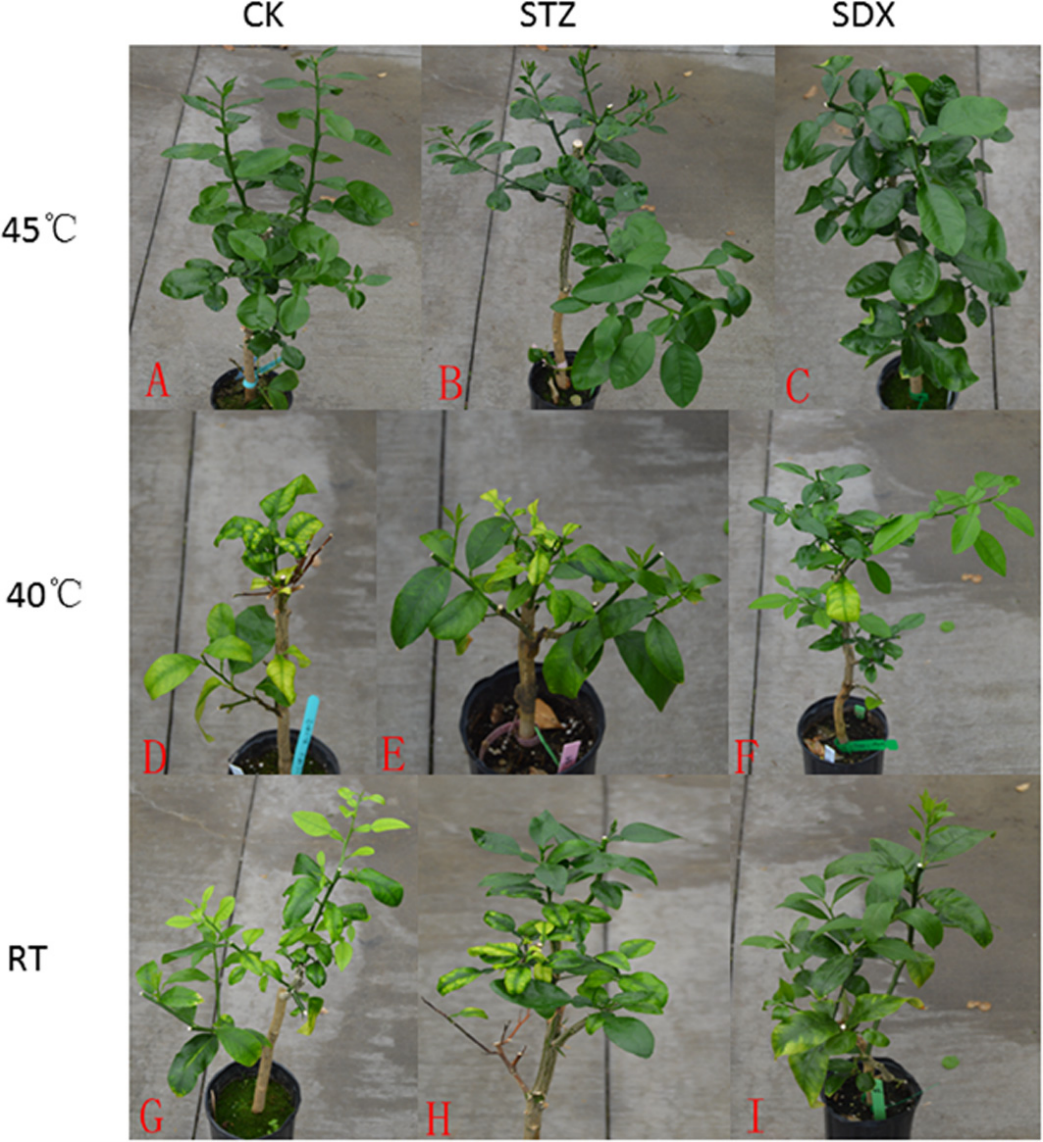
HLB-affected citrus treated with thermo-chemotherapy. **A:** Thermotherapy at 45°C; **B:** Combination of thermotherapy at 45°C and chemotherapy with STZ; **C:** Combination of thermotherapy at 45°C and chemotherapy with SDX; **D:** Thermotherapy at 40°C; **E:** Combination of thermotherapy at 40°C and chemotherapy with STZ; **F:** Combination of thermotherapy at 40°C and chemotherapy with SDX; **G:** Without thermotherapy and chemotherapy (Kept at room temperature—RT); **H:** Chemotherapy with STZ at room temperature (RT); **I:** Chemotherapy with SDX at room temperature (RT).

HLB-affected citrus were treated with combinations of thermotherapy at various temperatures (45°C, 40°C and room temperature) and chemotherapy with different chemical compounds (SDX, STZ and tap water (CK)) by root drench application. HLB-affected citrus treated with thermotherapy at 45°C recovered and the disease index (DI) was 0% at 300 days after initial treatment (Fig 1). On citrus treated with thermotherapy at 40°C, DI and Las titer remained high, as did the DI and Las titer of the citrus that received no thermotherapy (RT) (Fig 1 and Table 1). The HybScore of eOUT_28, which represents “*Candidatus* Liberibacter,” was lower in all the samples treated at 45°C than in samples treated at 40°C or RT (Table 1).

**Table 1:** Efficacy of thermo-chemotherapy against citrus HLB.

Temperature	Chemical compound	Disease index (%)				AUDPCs	Las bacterial titers^b^	Hybscore^c^
		Initial	60 DAT	180 DAT	300 DAT		300 DAT	300 DAT
45°C	SDX	100.00	0.00	0.00	0.00	11290.31±90.85a	226.99	87
	STZ	91.67	41.67	0.00	0.00	10667.91±637.29a	226.99	131
	Tap water(CK)	91.67	83.33	8.33	0.00	9687.32±463.19b	226.99	77
40°C	SDX	91.67	75.00	83.33	75.00	7017.62±308.48a	4164318.32	426
	STZ	100.00	58.33	75.00	75.00	7462.7±323.51a	2410599.98	357
	Tap water(CK)	83.33	50.00	91.67	75.00	7443.23±1084.33a	1667061.70	430
RT^a^	SDX	91.67	75.00	50.00	66.67	8240.28±1754.07a	685758.85	417
	STZ	100.00	100.00	83.33	50.00	6726.12±179.61a	511792.79	424
	Tap water(CK)	100.00	91.67	75.00	50.00	7377.93±335.11a	336697.55	457

^a^ RT indicated that HLB-affected citrus was maintained in a greenhouse at room temperature before chemotherapy.

^b^ Las bacterial titer is presented as # templates per gram of tissue.

^c^ Hybscore of eOTU_28, representing”*Candidatus* Liberibacter”.

All data were analyzed by Duncan’s multiple range test using SAS software package. Values denoted by the same letter (a or b) are not significantly different within the same treatment at *p* ≤ 0.05 level of significance.

Treatment with chemical compounds combined with thermotherapy at 40°C was not effective for combating HLB, nor was application of these chemical compounds alone (Table 1). Although thermotherapy at 45°C was effective against HLB, standardized area under disease progress curves (AUDPCs) of the STZ and SDX treatments combined with thermotherapy at 45°C were significantly higher than that of thermotherapy at 45°C alone (Table 1). Following thermotherapy at 45°C, the DIs of STZ and SDX treatments were zero at 60 days and 180 days after initial treatment, respectively, while DI of the citrus treated with tap water (CK), was zero at 300 days after initial treatment (Table 1).

### Bacterial microbiome in response to thermotherapy and chemotherapy

**Fig 2:**
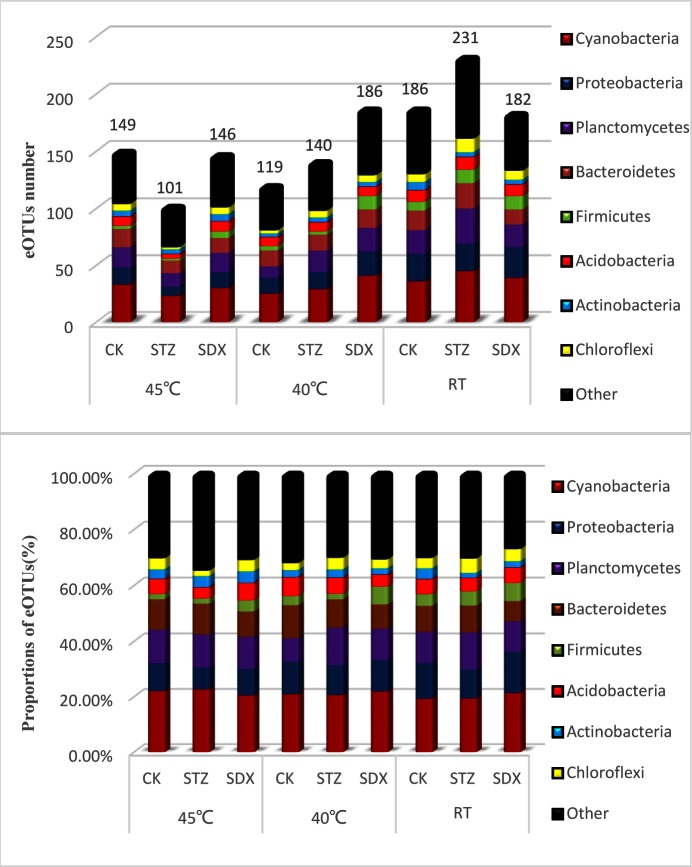
Relative proportions of eOTUs in the microbiome at the phylum level in HLB-affected citrus treated with combination of thermotherapy at 45°C, 40°C, and room temperature and chemotherapy with tap water, STZ and SDX.

A total of 311 different eOTUs were detected in midribs from all tested citrus plants, of which 42 OTUs (13.50%) were shared by all samples. In total, 26 phyla were detected, of which 8 phyla had 10 or more eOTUs: *Cyanobacteria* (18.01%), *Proteobacteria* (13.50%), *Planctomycetes* (10.61%), *Bacteroidetes* (9.64%), *Firmicutes* (5.14%), *Acidobacteria* (5.14%), *Actinobacteria* (4.18%), and *Chloroflexi* (4.50%). *Cyanobacteria* were dominant in the bacterial populations after thermo-chemotherapy (Fig 2).

201 eOTUs (64.63%) were detected in all the samples with thermotherapy at 45°C, which is fewer than the number found in leaf samples treated with thermotherapy at 40°C (230 eOTUs, 73.96%) or without thermotherapy (282 eOTUs, 90.68%) (S1 Fig). In citrus leaves not receiving thermotherapy treatment or with thermotherapy at 40°C, the population of *Proteobacteria* was lower than in leaves receiving thermotherapy at 45°C (Fig 2 and S1 Table). Members of class *Alphaproteobacteria* decreased significantly, and no eOTUs representing the genus *Candidatus* Liberibacter were detected in leaf samples from the citrus treated with STZ and SDX combined with thermotherapy at 45°C (S1 Table). In addition, Shannon’s and Simpson’s diversity indices both revealed greater family diversity in all the chemical treatments without thermotherapy (Table 2), indicating that thermotherapy results in reduced microbiome diversity. Thus, under the tap water treatment, thermotherapy at 45°C increased the number of eOTU in the phyla of *Actinobacteria*, *Planctomycetes* and *Cyanobacteria* (S1 Table), which was resulted to abundant bacterial families were detected in tap water treatment combined with thermotherapy at 45°C, compared to tap water treatment combined with thermotherapy at 40°C (Table 2).

**Table 2:** Bacterial families detected and biodiversity indices in the HLB-affected citrus treated with thermo-chemotherapy.

Temperature	Chemical compounds	Family detected	Simpson’s index (D)	Shannon’s index (H)
45°C	CK	71	0.969	3.900
	STZ	50	0.957	3.610
	SDX	69	0.960	3.815
40°C	CK	57	0.962	3.718
	STZ	69	0.968	3.879
	SDX	83	0.968	3.988
RT	CK	92	0.972	4.103
	STZ	95	0.969	4.049
	SDX	84	0.970	4.048

The PhyloChip^TM^G3 data also indicated a change in the microbial community following chemical treatments. 255 eOTUs (81.99%) and 260 eOTUs (83.60%) were detected in the STZ and SDX treatments, respectively, and these values were higher than the tap water (CK) treatment (238 OTUs, 76.52%) (S1 Fig). *Firmicutes* diversity increased in leaf samples from the citrus treated by SDX, compared with STZ and CK treatment. In particular, a greater number of eOTU of class *Clostridia* belonging to *Firmicutes* were detected in the SDX treatment (Fig 2 and S1 Table). The effect of SDX and STZ on population diversity of several phyla was different depending on thermotherapy at various temperatures. The proportion of *Proteobacteria*, *Planctomycetes*, and *Bacteroidetes* decreased in leaf sample from citrus treated at 45°C and STZ or SDX, in comparison to thermotherapy at 45°C alone (Fig 2). No eOTUs in the *Rhizobiaceae* family, which belongs to the *Proteobacteria*, were detected in leaf samples of the citrus in STZ or SDX treatment combined with thermotherapy at 45°C, and only one eOTU in the *Rhizobiaceae* family was detected in tap water treatment (CK) combined with thermotherapy at 45°C (S1 Table). Thus, the proportion of these bacterial phyla fluctuated in the chemical treatments combined with thermotherapy at 40°C and chemical treatments alone (Fig 2). Furthermore, Shannon’s and Simpson’s diversity indices both revealed that under thermotherapy at 45°C, lower levels of family diversity were present in response to STZ and SDX treatment (Table 2), compared to tap water treatment (CK).

### Specific OTUs associated with thermotherapy and chemotherapy

**Fig 3:**
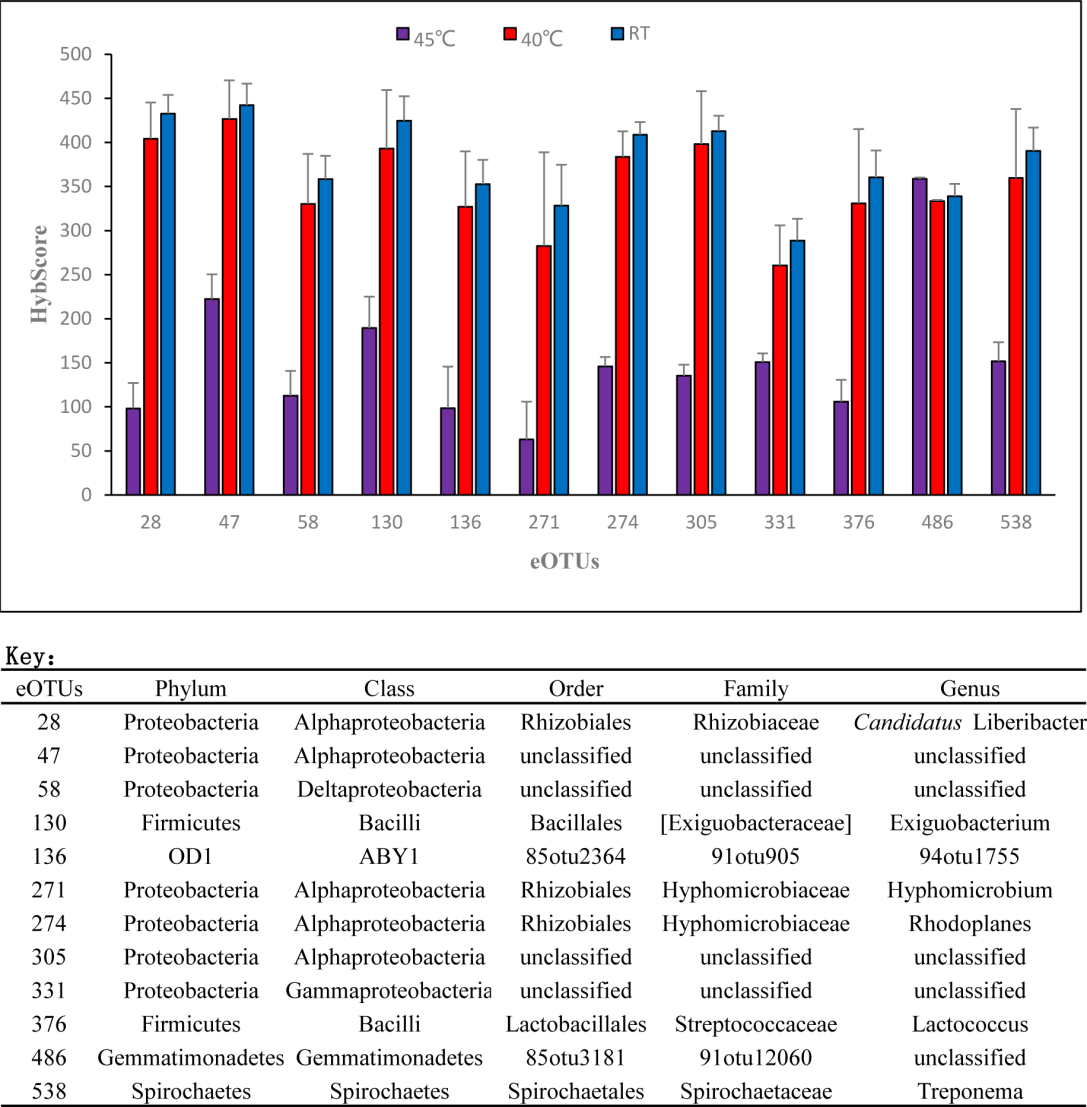
Phylochip^TM^G3 Hybscore for 12 of 48 taxa selected by Filter-5 in the HLB-affected citrus treated with thermotherapy at 45°C, 40°C or room temperature (RT). These 12 taxa were significantly different (*P* < 0.05) in response to thermotherapy. The error bars indicate standard errors.

Principal coordinate analysis (PCoA) based on weighted Unifrac distances between samples was performed with the PhyloChip^TM^G3 community data sets, and the results indicated that there were significant differences among thermotherapy and chemotherapy treatments. The 48 eOTUs were selected with Filter-5. We separately examined the eOTUs that were significantly different (*p* < 0.05) between the various thermotherapy regimens. Only 12 eOTUs (eOTU_28, eOTU_47, eOTU_58, eOTU_130, eOTU_136, eOTU_271, eOTU_274, eOTU_305, eOTU_331, eOTU_376, eOTU_486, and eOTU_538) were significantly different. Of these, 7 eOTUs were from *Proteobacteria*, 2 eOTUs were from *Firmicutes*, 1 eOTU was from *Spirochaetes* and 1 eOTU was from *OD1*. eOTU_486 had a lower HybScore in the thermotherapy treatment at 45°C compared with thermotherapy at 40°C or without thermotherapy (RT) (Fig 3). The Hybscore (98.33 ± 28.73) value of eOTU_28 (‘*Ca*. Liberibacter’) resulting from thermotherapy at 45°C, was significantly lower than that of the thermotherapy treatment at 40°C or without thermotherapy, which agrees well with the measured Las titers. Moreover, abundance of eOTU_486, which belongs to phylum *Gemmatimonadetes*, was increased by thermotherapy at 45°C (Fig 3).

**Fig 4:**
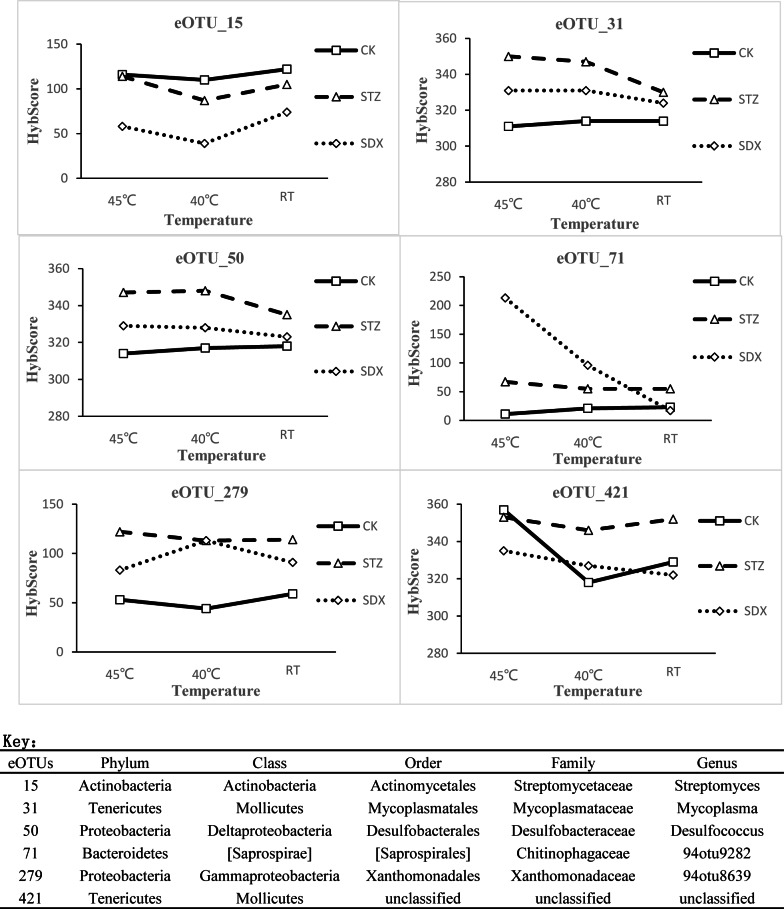
Phylochip^TM^G3 Hybscore for six specific operational taxon units (eOTUs) in leaf samples of HLB-affected citrus treated with different chemicals (Tap water (CK), STZ, and SDX) combined with thermotherapy at various temperatures (RT, 40°C and 45°C).

The 6 eOTUs (eOTU_15, eOTU_31, eOTU_50, eOTU_71, eOTU_279, and eOTU_421) were selected by Filter-5. These eOTUs were significantly different (*p* < 0.05) in response to chemotherapy with STZ or SDX. The Hybscores of eOTU_31, eOTU_50 and eOTU_279 receiving no chemical treatment (CK) were lower than those resulting from STZ and SDX treatments, while the abundance of eOTU_15 was greater in CK (Fig 4). The HybScore of eOTU_71 in the SDX treatment increased significantly following thermotherapy at 45°C (Fig 4). Compared with SDX and STZ treatments, HybScore of eOTU_421 in the CK treatment varied widely with different thermotherapy regimens (Fig 4).

## Discussion

HLB is disaster disease for citrus production. In previous studies, it was reported that Las progression is related to change of microbial community in HLB-affected citrus [16, 17]. In this study, the combination of heat treatment at 45°C and sulfonamide antibiotics can effectively suppress Las and affect microbial community, and microbial community in HLB-affected citrus following thermotherapy and chemotherapy was deciphered by microarray analysis.

Using the PhyloChip^TM^G3 array, a total of 311 eOTUs in 26 phyla were detected in leaf midribs of HLB-affected citrus trees after thermotherapy at various temperatures and chemotherapy with sulfonamide antibiotics (STZ and SDX). The PhyloChip^TM^G3 array is capable of detecting 50,000 bacterial taxa using a high-specificity probe set. Previously, 7,028 OTUs in 58 phyla were detected in citrus leaf midribs in HLB-affected, field-grown citrus treated with PS (Penicillin G and Streptomycin) and KO (Kasugamycin and oxytetracyline) antibiotic regimens [16]. In our current study, the bacterial microbiome of citrus plants were characterized in response to Las infection and treatments with ampicillin (Amp) and gentamycin (Gm) by PhyloChip^TM^G3-based metagenomics. 7,407 OTUs in 53 phyla were detected in citrus leaf midribs [21]. Responsive probes were grouped into “probe sets” empirically based on both correlations in fluorescence intensities across all biological samples and the taxonomic relatedness of the probe sequences. This is a more conservative method for annotating taxa and reduces the likelihood of diversity overestimation. We then annotated empirical OTUs (eOTUs) according to a recently described taxonomic hierarchy [31]. This method can limit the erroneous identification of OTUs. Our results demonstrated that *Cyanobacteria* was the dominant phylum, followed by *Proteobacteria*, *Planctomycetes*, *Bacteroidetes*, *Firmicutes*, *Acidobacteria*, *Actinobacteria*, and *Chloroflexi*. These results are not consistent with previous studies in that *Proteobacteria* is a dominant phylum in HLB-affected citrus treated by ampicillin (Amp), gentamycin, a combination of Penicillin G and Streptomycin) and a combination of Kasugamycin and oxytetracyline [16, 21]. However, heat and sulfonamide treatment may result in different changes in the bacterial community of HLB-affected citrus, favoring the proliferation of *Cyanobacteria*.

In this study, thermotherapy at 45°C was more effective against Las than thermotherapy at 40°C or RT (without thermotherapy). 300 days after initial treatment, no Las was detected in HLB-affected citrus treated with thermotherapy at 45°C (Table 1 and Fig 1). The efficacy of thermotherapy against Las depends upon the duration of treatment, temperature of treatment, and citrus varieties [11]. Due to different duration of heat treatment and citrus varieties, the temperature at 45°C against Las in this study is different from previous studies, which demonstrated that 35°C or 40°C can suppress Las titer in citrus tissue [11, 12]. In addition, due to Las residing in phloem as well as poor condition of root system of HLB-affected citrus [10], it has been difficult to deliver effective compounds against Las into citrus phloem through root. In this study, thermotherapy at 45°C can also enhance the vigor of citrus and can promote root growth, for improving delivery efficiency of sulfonamide antibiotics into citrus phloem against Las through root. However, when sulfonamide antibiotics were applied to HLB-affected citrus trees by root drench without heat treatment or heat treatment at 40°C, the Las titer was unaffected. This finding may have been due to low absorption and utilization of the chemical compounds.

Understanding the structure and species composition of bacterial communities is necessary to evaluate the influence of the applied temperature treatment. Temperature is a key factor regulating the activity and determining the composition of a microbial community. If the temperature changes, a selection pressure will be applied, altering the microbial community. Several previous studies demonstrated that after heat treatment, the population of microorganisms immediately decreases [33, 34]. The bacterial diversity and eOTUs of leaf samples from HLB-affected citrus treated with thermotherapy at 45°C were decreased. This is particularly true of the eOTUs in the *Proteobacteria* phylum (Fig 2), since 7 of 12 specific eOTUs which were significantly different in response to thermotherapy belonged to this phylum. The HybScores of these 7 eOTUs following thermotherapy at 45°C were lower than in the thermotherapy treatments at 40°C or RT (without thermotherapy). eOTU_28, representing “*Candidatus* Liberibacter,” the putative causal agent of citrus HLB [17], had a lower HybScore with thermotherapy at 45°C. The HybScore of the other 6 specific eOTUs belonging to *Proteobacteria* (eOTU_47, eOTU_58, eOTU_271, eOTU_274, eOTU_305, and eOTU_331), 2 specific eOTUs belonging to *Firmicutes* (eOTU_130 and eOTU_376), eOTU_136 belonging to *OD1*, and eOTU_538 belonging to *Spirochaetes* were also significantly decreased by thermotherapy at 45°C (Fig 3). These HybScore reductions with thermotherapy at 45°C may be related to secondary proliferation in declining leaves rather than relating to initial HLB development. However, the HybScore of eOTU_486 belonging to class *Gemmatimonadetes* with thermotherapy at 45°C was higher than in the thermotherapy treatment at 40°C or RT. The *Gemmatimonadetes* possess a pathway which is related to the biosynthesis of tetracycline [35] and tetracycline is an antibiotic that can suppress HLB [36, 37]. Therefore, the abundance of *Gemmatimonadetes* may contribute to suppression of HLB. *Gemmatimonadetes* also is the bacterial phylum containing chlorophyll-based phototrophic species [38]. Abundant *Gemmatimonadetes* may enhance photosynthetic efficiency [38], which may enhance the vigor of citrus and increase delivery efficiency of effective chemical compounds to combat HLB. In addition, several bacterial OTUs from the phyla of *Actinobacteria*, *Planctomycetes* and *Cyanobacteria* can survive at high temperature [39, 40]. This study demonstrated that the eOTUs in these bacterial phyla were more abundant in tap water treatment combined with thermotherapy at 45°C, compared to tap water treatment combined with thermotherapy at 40°C (Table 2). Therefore, these heat-tolerance bacteria may be related to induce defense system against Las or enhance the vigor of citrus.

Sulfonamide antibiotics (STZ and SDX) are organic sulfur compounds containing the radical SO_2_NH_2_ (the amides of sulfonic acids). Their molecular structure is similar to p-aminobenzoic acid (PABA), a substrate of the enzyme dihydropteroate synthetase required for the synthesis of tetrahydrofolic acid in bacteria [41, 42]. STZ and SDX were proven effective against Las by graft-based assay [8]. In this study, Sulfonamide antibiotics (STZ and SDX) combined with thermotherapy at 45°C were also effective in combating HLB, using root drench application. Our results also demonstrated that a combination of chemotherapy (SDX or STZ) and thermotherapy at 45°C was more effective against HLB than thermotherapy at 45°C alone by AUDPCs analysis (Table 1 and Fig 1). This finding suggests that thermotherapy may have contributed to alteration of the microbial community in response to SDX and STZ. Six specific eOTUs were found to be significantly different after chemical treatment. The HybScores of four eOTUs (eOTU_31, eOTU_50, eOTU_71 and eOTU_279) were enhanced by STZ and SDX treatment (Fig 4). eOTU_50 represents the genus *Desulfococcus* which contains reducing sulfide bacteria. These bacteria produce sulfur-rich proteins such as Thionins and Defensins [43]. These sulfur-rich proteins have antimicrobial activity and induce plant defense systems against plant pathogens [44, 45]. eOTU_71 belongs to the *Chitinophagaceae* family. It is known that some members of this family are chitinolytic [46]. Thereby, eOTU_71 may be associated with inhibiting the growth of plant pathogens through the production of chitinase. The abundance of eOTU_71 was greatest in the SDX treatment with thermotherapy at 45°C, which may have contributed to a higher antibacterial activity against the Las. eOTU_279 is from the family *Xanthomonadaceae*. It has been reported that several genera in this family can produce antifungal antibiotics and have growth promoting activities on plants [47]. The family *Actinomycetales* was abundant in leaves and roots of HLB-affected citrus [17, 18]. The HybScore of eOTU_15 from *Actinomycetales* in the tap water treatment (CK) was higher than in the STZ or SDX treatments, which may be related to HLB progress. *Mycoplasma* is a genus of bacteria that lack a cell wall around their cell membranes [48]. Without a cell wall, they are unaffected by many common antibiotics such as penicillin and sulfonamide [49]. In this study, eOTU_31 which is in genus *Mycoplasma*, was resistant to sulfonamide antibiotics (STZ and SDX) (Fig 4), but the mechanism through which the HybScore of eOTU_31 was enhanced by STZ and SDX is unknown. eOTU_421 belongs to class *Mollicutes*. This class was abundant in the tap water drench treatment with thermotherapy at 45°C and its abundance may be related to Las proliferation in citrus. In addition, our results also indicated greater eOTU in phylum *Firmicutes* in response to SDX treatment, and eOTU in class *Clostridia* were particularly abundant. In a previous study, *Clostridia* contributed to plant growth, including root growth [50, 51].

In summary, our study characterized the microbial community structure in HLB-affected citrus treated with thermotherapy and chemotherapy with sulfonamide antibiotics (STZ and SDX). The change in microbial community has an association with HLB progression. Our research also provided an effective, integrated strategy using a combination of thermotherapy at 45°C and chemotherapy with SDX or STZ by root drench application for combating HLB. Moreover, we provide information for developing more effective and eco-friendly biocontrol strategies to combat HLB in the context of citrus production.

## Supporting Information

S1 Fig Empirical Operational Taxonomic Units (eOTU) numbers present. **A:** The eOTU numbers were detected in tap water treatment (CK). **B:** The eOTU numbers were detected in sulfathiazole sodium treatment (STZ). **C:** The eOTU numbers were detected in sulfadimethoxine sodium treatment (SDX). **D:** The eOTU numbers were detected in heat treatment at 45°C. **E:** The eOTU numbers were detected in heat treatment at 40°C. **F:** The eOTU numbers were detected at room temperature (RT). CK-45: Combination of thermotherapy at 45°C and tap water treatment (CK), CK-40: Combination of thermotherapy at 40°C and tap water treatment (CK), CK-RT: Without thermotherapy and chemotherapy (Kept at room temperature—RT). STZ-45: Combination of thermotherapy at 45°C and chemotherapy with STZ. STZ-40: Combination of thermotherapy at 40°C and chemotherapy with STZ. STZ-RT: Chemotherapy with STZ at room temperature (RT). SDX-45: Combination of thermotherapy at 45°C and chemotherapy with SDX. SDX-40: Combination of thermotherapy at 40°C and chemotherapy with SDX. SDX-RT: Chemotherapy with SDX at room temperature (RT).
(TIF)

S1 Table Total number of empirical Operational Taxonomic Units (eOTUs) in three replicates (n = 3) detected by PhyloChip™ G3 hybridization in leaf midribs from HLB-affected citrus treated with thermo-chemotherapy. (DOCX)
