# Supplementary figures and images for: Deciphering the Bacterial Microbiome in Huanglongbing-Affected Citrus Treated with Thermotherapy and Sulfonamide Antibiotics
Source: PLoS One. 2016 May 12;11(5):e0155472. doi: 10.1371/journal.pone.0155472 (PMC4865244; doi:10.1371/journal.pone.0155472)

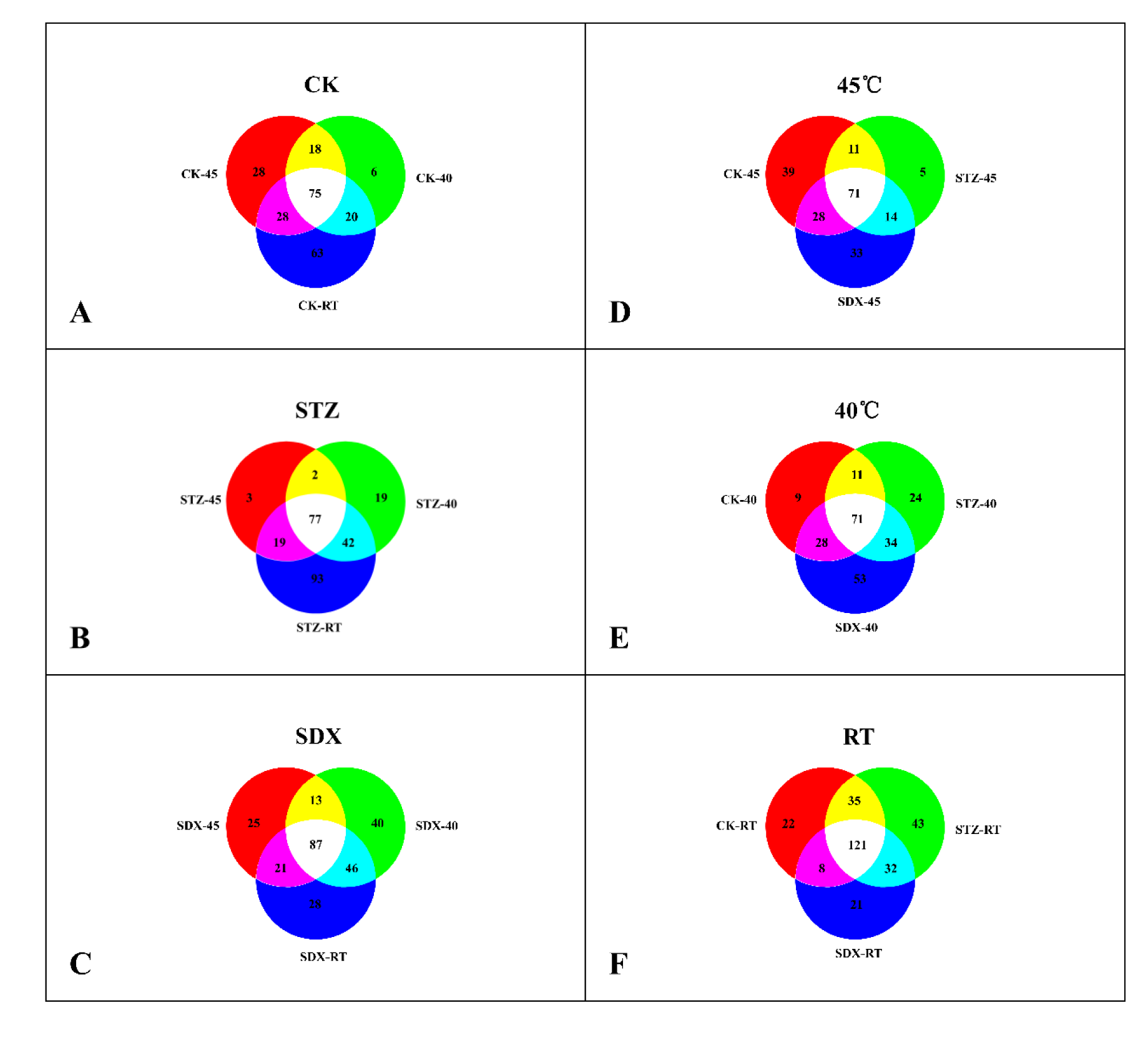

Supplement: S1 Fig — A: The eOTU numbers were detected in tap water treatment (CK). B: The eOTU numbers were detected in sulfathiazole sodium treatment (STZ). C: The eOTU numbers were detected in sulfadimethoxine sodium treatment (SDX). D: The eOTU numbers were detected in heat treatment at 45°C. E: The eOTU numbers were detected in heat treatment at 40°C. F: The eOTU numbers were detected at room temperature (RT). CK-45: Combination of thermotherapy at 45°C and tap water treatment (CK), CK-40: Combination of thermotherapy at 40°C and tap water treatment (CK), CK-RT: Without thermotherapy and chemotherapy (Kept at room temperature—RT). STZ-45: Combination of thermotherapy at 45°C and chemotherapy with STZ. STZ-40: Combination of thermotherapy at 40°C and chemotherapy with STZ. STZ-RT: Chemotherapy with STZ at room temperature (RT). SDX-45: Combination of thermotherapy at 45°C and chemotherapy with SDX. SDX-40: Combination of thermotherapy at 40°C and chemotherapy with SDX. SDX-RT: Chemotherapy with SDX at room temperature (RT). (TIF) [file pone.0155472.s001.tif]
