# Supplementary material for: Deciphering the Bacterial Microbiome in Huanglongbing-Affected Citrus Treated with Thermotherapy and Sulfonamide Antibiotics
Source: PLoS One. 2016 May 12;11(5):e0155472. doi: 10.1371/journal.pone.0155472 (PMC4865244; doi:10.1371/journal.pone.0155472)
Supplement: S1 Table — (DOCX) [file pone.0155472.s002.docx]

**Appendix**

**S1 Table.** Total number of empirical Operational Taxonomic Units (eOTUs) in three replicates (n=3) detected by PhyloChip™ G3 hybridization in leaf midribs from HLB-affected citrus treated with thermo-chemotherapy

| **Kingdom** | **Phylum** | **Class** | **Order** | **Family** | **Thermotherapy at 45℃** | | | **Thermotherapy at 40℃** | | | **Room Temperature(RT)** | | |
| --- | --- | --- | --- | --- | --- | --- | --- | --- | --- | --- | --- | --- | --- |
|  |  |  |  |  | **CK** | **STZ** | **SDX** | **CK** | **STZ** | **SDX** | **CK** | **STZ** | **SDX** |
| *Bacteria* | *Acidobacteria* |  |  |  | 8 | 4 | 9 | 8 | 8 | 8 | 10 | 11 | 10 |
|  |  | *Acidobacteria-6* | *iii1-15* | *91otu412* | 5 | 2 | 3 | 3 | 3 | 4 | 3 | 5 | 4 |
|  | *Actinobacteria* |  |  |  | 5 | 4 | 6 | 3 | 4 | 4 | 7 | 4 | 4 |
|  |  | *Actinobacteria* | *Actinomycetales* |  | 5 | 2 | 3 | 1 | 2 | 2 | 4 | 2 | 2 |
|  |  |  |  |  |  |  |  |  |  |  |  |  |  |
|  | *Bacteroidetes* |  |  |  | 16 | 11 | 13 | 14 | 14 | 16 | 17 | 22 | 13 |
|  |  | *Bacteroidia* |  |  | 13 | 8 | 12 | 11 | 11 | 10 | 13 | 17 | 9 |
|  |  |  | *Bacteroidales* |  | 13 | 8 | 12 | 11 | 11 | 10 | 13 | 17 | 9 |
|  |  |  |  | *Porphyromonadaceae* | 4 | 3 | 2 | 4 | 5 | 6 | 7 | 6 | 4 |
|  | *Chloroflexi* |  |  |  | 6 | 2 | 6 | 3 | 6 | 6 | 7 | 12 | 8 |
|  |  | *Anaerolineae* |  |  | 6 | 2 | 6 | 3 | 6 | 6 | 7 | 12 | 8 |
|  | *Cyanobacteria* |  |  |  | 33 | 23 | 30 | 25 | 29 | 41 | 36 | 45 | 39 |
|  |  | *Chloroplast* |  |  | 30 | 22 | 29 | 24 | 27 | 39 | 34 | 43 | 36 |
|  |  |  | *Streptophyta* |  | 17 | 15 | 23 | 17 | 15 | 24 | 21 | 27 | 23 |
|  |  |  |  | *91otu7296* | 15 | 14 | 21 | 15 | 14 | 22 | 19 | 25 | 21 |
|  |  |  | *Chlorophyta* |  | 6 | 3 | 5 | 3 | 4 | 5 | 5 | 6 | 4 |
|  | *Firmicutes* |  |  |  | 3 | 2 | 6 | 4 | 3 | 12 | 8 | 12 | 12 |
|  |  | *Clostridia* |  |  | 3 | 2 | 6 | 4 | 3 | 10 | 7 | 12 | 9 |
|  |  |  | *Clostridiales* |  | 3 | 2 | 6 | 4 | 3 | 10 | 7 | 12 | 9 |
|  | *Planctomycetes* |  |  |  | 18 | 12 | 17 | 10 | 19 | 21 | 21 | 31 | 20 |
|  |  | *Planctomycetia* |  |  | 11 | 7 | 11 | 6 | 10 | 11 | 13 | 18 | 10 |
|  |  |  | *Pirellulales* |  | 8 | 4 | 8 | 4 | 8 | 9 | 10 | 14 | 7 |
|  |  |  |  | *Pirellulaceae* | 8 | 4 | 8 | 4 | 8 | 9 | 10 | 14 | 7 |
|  |  | *Phycisphaerae* |  |  | 4 | 4 | 5 | 2 | 7 | 7 | 5 | 10 | 8 |
|  | *Proteobacteria* |  |  |  | 15 | 8 | 14 | 14 | 15 | 21 | 24 | 24 | 27 |
|  |  | *Alphaproteobacteria* |  |  | 4 | 1 | 2 | 6 | 2 | 8 | 11 | 9 | 10 |
|  |  |  | *Rhizobiales* |  | 1 | 0 | 0 | 1 | 1 | 3 | 2 | 3 | 1 |
|  |  | *Gammaproteobacteria* |  |  | 6 | 4 | 4 | 3 | 5 | 4 | 5 | 5 | 7 |
|  | *Tenericutes* |  |  |  | 5 | 5 | 4 | 3 | 5 | 7 | 6 | 7 | 5 |
|  |  | *Mollicutes* |  |  | 5 | 5 | 4 | 3 | 5 | 7 | 6 | 7 | 5 |
|  |  |  | *Mycoplasmatales* | *Mycoplasmataceae* | 3 | 3 | 3 | 2 | 4 | 5 | 5 | 5 | 4 |
| Other |  |  |  |  | 40 | 30 | 41 | 35 | 37 | 50 | 50 | 63 | 44 |
| Total |  |  |  |  | 149 | 101 | 146 | 119 | 140 | 186 | 186 | 231 | 182 |
